# Growth hormone treatment for adults with Prader-Willi syndrome: another point of view

**DOI:** 10.1186/s13023-021-01952-9

**Published:** 2021-08-03

**Authors:** Harry J. Hirsch, Varda Gross-Tsur

**Affiliations:** 1 Israel Multidisciplinary Prader-Willi Syndrome Clinic, Neuropediatric Unit, Department of Pediatrics, Shaare Zedek Medical Center, Jerusalem, Israel; 2 The Faculty of Medicine, The Hebrew University School of Medicine, Jerusalem, Israel

**Keywords:** Prader-Willi syndrome, Growth hormone, Obesity, Insulin-like growth factor-1

## Abstract

Growth hormone treatment for children with Prader Willi syndrome (PWS) has shown proven benefits not only in increasing final height but also with positive effects on body composition and motor development. In a recent letter to the editor, Hoybye and colleagues recommend growth hormone treatment for adults with PWS based exclusively on the genetic diagnosis and without regard for growth hormone secretory status. We question whether the benefits of growth hormone treatment in PWS adults, mainly improvement in body composition, are significant enough to justify the as yet unkown consequences of long-term treatment in an adult population. Morbidity and mortality in PWS are mainly due to complications of obesity, and growth hormone treatment does not result in a decrease in BMI or waist circumference. Increases in insulin-like factor-1 as a result of growth hormone treatment over the course of several decades in PWS adults raises concern over possible increase risk of cancer. Compliance with daily injections is likely to be poor. We suggest that efforts to provide appropriate dietary and exercise regimens may be more beneficial and cost-effective than advocating for growth hormone treatment for adults with PWS.

Growth hormone treatment for adults with PWS has been shown to improve body composition, increase muscle strength and endurance, and result in modest improvements in cognition as summarized in the letter to the editor by Hoybe, Holland, and Driscoll [1]. The authors recommend growth hormone treatment for all adults with a genetically confirmed diagnosis of PWS, irrespective of their responses to standard growth hormone stimulation tests or whether they had previously received growth hormone during childhood. Despite some proven benefits of growth hormone treatment, we question, for several reasons, the recommendation to advocate for growth hormone treatment of all PWS adults.

Improvements in body composition associated with GH (growth hormone) treatment, mainly a decrease in visceral adipose tissue (VAT) and increase in lean body mass, may not necessarily result in significant improvements in health or quality of life. PWS individuals are protected to some degree from the adverse metabolic effects of VAT. Fatty liver is rare in PWS and PWS individuals have less insulin resistance compared to individuals with non-syndromic obesity [2–5]. Nutritional intervention even without growth hormone treatment has been shown to maintain VAT within the normal reference range [6]. Morbidity and mortality in PWS result primarily from obesity-related complications and hyperphagia, such as respiratory abnormalities, choking, aspiration, gastric or intestinal perforations or obstructions and as Hoybe and colleagues stated, GH treatment does not result in decreases in BMI (body mass index) or waist circumference [1, 7].

While GH treatment for adults with severe GH deficiency (mostly due to organic causes such as CNS tumors or congenital hypopituitarism) has been shown to improve quality of life, most PWS adults do not have severe GH deficiency. GH deficiency in PWS adults appears to be variably mild or partial [8–10]. We are not aware that there is any evidence for long-term benefit of GH treatment for adults who have only mild or partial GH deficiency.

Hypogonadism is a common feature of PWS and contributes to the abnormal body composition in this syndrome [11, 12]. Androgen replacement improves lean body mass, is less expensive and is more likely to be adhered to than GH injections [13].

Poor compliance with daily growth hormone injections is common in children [14]. For PWS adults, compliance is likely to be poor—at least until, as the authors point out, once-weekly GH preparations become available. Furthermore, GH treatment is expensive and recommending growth hormone for all PWS adults might sidetrack limited resources (family finances and government funding) from more basic effective aspects of PWS management such as nutritional management, exercise programs, and opening hostels designated specifically for PWS adults [15]. Pellikan et al. reported that even within the framework of a major multidisciplinary PWS clinic, 22% of the adults exercised too little and 37% were not being seen by a dietitian [12]. Addressing these more basic issues may have a greater impact on health and quality of life than might be achieved by GH treatment.

Widespread use of GH could result in inappropriate use of growth hormone in individuals with severe obesity for whom GH treatment may significantly increase obstructive sleep apnea and/or hyperglycemia. Although growth hormone treatment for children and adolescents has a proven safety record, there may be some concerns about long-term treatment of older adults with PWS. Of particular concern is the effect of “normalizing” the relatively low serum insulin-like growth factor-1 (IGF-1) levels usually seen in PWS individuals. Patients with low IGF-1 levels due to congenital growth hormone deficiency or growth hormone insensitivity (e.g. Laron-type dwarfism) have significantly lower incidences of cancer, while individuals with acromegaly and higher than average levels of insulin-like growth factor-1 (IGF1) have an increased risk of cancer [16, 17]. The incidence of colorectal cancer is significantly greater for individuals with IGF-1 levels in the upper range of normal compared to those with levels in the lower range [18]. Similar population studies have shown associations with IGF-1 levels for prostate, breast, and lung cancer [19]. IGF-1 levels are usually low in most PWS individuals [20]. Even a modest increase in IGF-1 due to GH treatment might lead to an increase risk of cancer in this population.

In a recent review of published studies of growth hormone treatment in adults with PWS, treatment duration was only one to two years in most reports, while only two described treatment durations of up to five years [21]. We are not aware of any studies which describe long-term benefits or possible adverse effects during several decades of treatment in PWS adults.

GH treatment for infants and children with PWS has shown clear beneficial effects on growth, development, and body composition and has a reassuring safety record over a typical duration of 10 to 15 years of daily administration. Some PWS adults, especially those individuals who are already receiving optimal dietary management and exercise programs, may also experience some benefit from GH treatment. Nevertheless, we suggest that further long-term studies of efficacy and safety are needed before recommending life-long GH treatment as the standard of care for all PWS adults.

## Data Availability

Data sharing is not applicable to this article as no datasets were generated or analysed during the current study.

## Abbreviations

PWS: Prader-Willi syndrome
GH: Growth hormone
VAT: Visceral adipose tissue
BMI: Body mass index = weight in kilograms/(height in meters)^2^
IGF-1: Insulin-like growth factor-1
