# Supplementary material for: Growth hormone treatment for adults with Prader-Willi syndrome: another point of view
Source: Orphanet J Rare Dis. 2021 Aug 3;16:337. doi: 10.1186/s13023-021-01952-9 (PMC8330048; doi:10.1186/s13023-021-01952-9)
Supplement: Related texts

# Reply: Response to Harry Hirsch and Varda Gross-Tsur: Growth hormone treatment for adults with Prader-Willi syndrome: another point of view

**Authors:** Charlotte Höybye, Anthony J. Holland, Daniel J. Driscoll, The Clinical, Scientific Advisory Board of The International Prader-Willi Syndrome Organisation

**Affiliations:** 3 grid.4714.6 0000 0004 1937 0626 Department of Endocrinology, Karolinska University Hospital and Department of Molecular Medicine and Surgery, Karolinska Institute, Stockholm, Sweden; 4 grid.5335.0 0000000121885934 Department of Psychiatry, University of Cambridge, Cambridge, UK; 5 grid.15276.37 0000 0004 1936 8091 Division of Pediatric Genetics and Metabolism, Department of Pediatrics, The John T. & Winifred M. Hayward Professor of Genetics Research, Chair, Prader-Willi Syndrome Association (USA) Clinical Advisory Board, Chair, International Prader-Willi Syndrome Organization Clinical & Scientific Advisory Board, University of Florida College of Medicine, Box 100296, Gainesville, FL 32610-0296 USA

In the letter by Dr. Hirsch and Dr. Gross-Tsur our advocacy for a general approval of growth hormone (GH) treatment of all adults with Prader-Willi syndrome (PWS) is questioned [22]. Their main arguments against our recommendation are that GH treatment has only been proven to have modest benefits and that the long-term effects and safety of GH treatment in adults with PWS are unknown.

Dr. Hirsch and Dr. Gross-Tsur argue that improvements in body composition associated with GH treatment are mainly due to a decrease in visceral adipose tissue (VAT) and an increase in lean body mass, which may not result in significant improvements in health or quality of life. They go on to argue that fatty liver is rare in PWS and insulin resistance is not pronounced, which to some degree protects people with PWS from the adverse metabolic effects of VAT, also referring to one study where intense nutritional intervention even without GH treatment was shown to maintain VAT within the normal reference range. Another argument they put forward is that morbidity and mortality are primarily caused by obesity-related complications secondary to hyperphagia and GH treatment does not result in decrease in BMI (body mass index) or waist circumference. In addition, Dr. Hirsch and Dr. Gross-Tsur point out that GH deficiency in PWS adults appears to be mild or partial and evidence for long-term benefit of GH treatment for adults who have only mild or partial GH deficiency is lacking.

As stated in our letter “Time for a general approval of growth hormone treatment in adults with Prader-Willi syndrome”, the hypothalamic dysfunction in PWS is permanent by nature, and therefore affected adults are expected to have the same hormone deficiencies as seen in the children [23]. GH deficiency has been for a long time accepted as part of the phenotype PWS, but a multitude of confounding factors are present and in PWS a different interpretation of the results from GH stimulation tests to those considered relevant in the typical population with GH deficiency is necessary. Therefore, it is not meaningful to grade GH deficiency in PWS or state that it is severe, mild, or partial.

Several studies of children with PWS have all shown improvements in height, body composition, cognitive function, and socialization [24, 25]. The number of studies in adults with PWS are less, but they have consistently shown improvements in body composition, physical performance, and psycho-social function [24–26]. The reduction in body fat and the increase in lean body mass are comparable to the effects of GH treatment in adults without PWS who fulfill criteria for profound GH deficiency [27]. GH’s lipolytic effect is most pronounced in VAT, but studies of adults with PWS have also shown a decrease in subcutaneous fat tissue [28]. We strongly agree that GH is not a weight reducing drug and moreover it does not affect hyperphagia [24, 25]. GH, or androgen treatment for that matter, does not replace a strictly controlled diet or regular physical exercise. Importantly, a strict diet and physical exercise can potentiate the effects of GH on body composition, metabolic factors, and general well-being.

The use of GH treatment in grossly obese patients is not recommended in any guideline. However, this is primarily not because of the risk of side effects, but because of lack of effect. We do not agree with the concerns about a potential increase in sleep apnea and hyperglycemia with GH treatment. One of us (CH) has recently published a study on GH treatment over three years in adults with PWS and an increase in sleep apnea was not seen [29]. In addition, previous studies have not shown an increase in the number of people with PWS with diabetes during GH treatment [25, 26]. In contrast, the development of diabetes has been found to be related to heredity and weight increase, independent of GH treatment [30]. GH is not widely used in adults with PWS whereas a strict diet and regular physical training are baseline treatments for all adults with PWS. It is well-known that people with PWS have problems complying to both diet and to physical training, and we do not disagree that it is of utmost importance to improve the implementation of these. Despite the fact that all are on a strict diet and regular physical activity obesity remains a large problem.

We have a long experience of treatment with GH in PWS and compliance with the GH injections is not a big issue. However, due to the behavioral problems in PWS the daily injections can be a burden for caregivers, and we hope the weekly injections will be an improvement. Some of us have treated adults with PWS for more than three decades and we have not observed an increase in diabetes or cancer related to GH treatment. Indeed, PWS is a rare disease and we acknowledge that the long-term consequences of GH treatment are not completely known, as is also the case with GH treatment of adults with GH deficiency of other etiologies, which is a considerably larger but still a rare group of patients [31]. The available data suggests that long-term GH treatment does not increase the risk of developing diabetes or malignancies [31]. We do not know of any appropriate group of patients for comparison with adults with PWS, but find the comparison with Laron dwarfs only theoretically relevant. Likewise, patients with acromegaly have long-term GH and insulin-like growth factor-1 (IGF1) levels far above the levels obtained with GH treatment and it has not been consistently proven that the risk of cancer is increased in people with acromegaly [31]. In our view it is not appropriate to generalize from the results from the general population with a different risk factor profile for cancer to people with PWS. An improvement in quality of life with GH treatment in adults with PWS has been shown, but as anybody working with people with PWS knows, adequate and validated measures for quality of life in PWS do not exist.

If the studies on GH treatment in PWS are few it should be noted that there is only one study on testosterone treatment in adults with PWS [32]. This study showed that low dose testosterone improved body composition without significant impairments in behavior [32]. However, the long-term potential negative effects of androgen treatment in men with PWS are also unknown. Despite this Dr. Hirsch and Dr. Gross-Tsur advocate for treatment of hypogonadism as androgens replacement improves lean body mass, is less expensive and the argue that adherence is likely to be better than to daily GH injections. Further, there are no studies of treatment with sex-steroids in women with PWS and the question is how Dr. Hirsch and Dr. Gross-Tsur think they should be treated.

The costs for GH treatment are high, but adults with PWS do not need high doses of GH and a common dose to adults above 30 years of age is 0.2–0.4 mg per day. In a rare disease, such as PWS, these costs would probably not reach the costs for multidisciplinary care and we would argue that its use together with good care brings additional benefits.

In summary, we do not agree with Dr. Hirsch and Dr. Gross-Tsur conclusions and still advocate for a general approval of growth hormone treatment in adults with Prader-Willi syndrome. We also still argue that GH treatment, with its many beneficial effects and few side effects, is a treatment that relieves some of the adverse effects of PWS in adults. Moreover, treatment with GH will improve the follow-up of adults with PWS as it has been shown for children with PWS. Whilst we are advocating for general approval of GH in adults with PWS, whether or not a person with PWS chooses to continue GH in adult life is for him/her to decide with guidance from those who support them. For some they may feel that the burden of the injections is too much, for others, they may be willing to accept this given the benefits that they have experienced. By ensuring that the principle of GH use in adults with PWS is established we make this choice possible as it is for those people with GH deficiencies for other reasons.
